# Rituximab and abatacept but not tocilizumab impair antibody response to pneumococcal conjugate vaccine in patients with rheumatoid arthritis

**DOI:** 10.1186/ar4358

**Published:** 2013-10-30

**Authors:** Meliha Crnkic Kapetanovic, Tore Saxne, Göran Jönsson, Lennart Truedsson, Pierre Geborek

**Affiliations:** 1 Department of Clinical Sciences Lund, Section of Rheumatology, Lund University, Lund, Sweden; 2 Department of Clinical Sciences Lund, Section of Infectious Diseases, Lund University, Lund, Sweden; 3 Department of Laboratory Medicine, Section of Microbiology, Immunology and Glycobiology, Lund University, Lund, Sweden

## Abstract

**Introduction:**

The objective of the study was to investigate the impact of newer biologic treatments including rituximab, abatacept and tocilizumab on antibody response following pneumococcal vaccination using a 7-valent conjugate vaccine in patients with established rheumatoid arthritis (RA).

**Methods:**

Patients with RA receiving rituximab, abatacept or tocilizumab as monotherapy or combined with methotrexate (MTX) participated in the study. Specific IgG antibodies against 23F and 6B serotypes were measured at vaccination and 4 to 6 weeks after vaccination using standardised ELISA. Geometric mean antibody levels (GML) were calculated. Antibody response (AR) was defined as the ratio between post- and pre-vaccination antibody levels and a positive antibody response (posAR) was AR ≥2.

**Results:**

In total, 88 patients were enrolled in the study. Of 55 patients treated with rituximab, 26 (46%) were on concomitant MTX. Of patients receiving abatacept (n = 17) and tocilizumab (n = 16) biologic treatment was given in combination with MTX in 13 (76%) and 9 (56%) patients, respectively. Patients treated with rituximab had significantly lower AR compared to those on tocilizumab, as well as compared to previously reported RA patients on MTX and controls (spondylarthropathy patients treated with NSAIDs and/or analgesics). In total, 10.3% of patients on rituximab monotherapy and no patient on rituximab + MTX had posAR for both serotypes. For abatacept and tocilizumab the corresponding figures were 17.6% and 50%.

**Conclusion:**

In this cohort of patients with established RA, treatment with rituximab and abatacept was associated with diminished antibody response but this was most pronounced for rituximab. Pneumococcal conjugate vaccine administrated during ongoing tocilizumab treatment seems to be associated with sufficient antibody response. Pneumococcal vaccination should preferably be encouraged before initiation of rituximab or abatacept treatment.

**Trial registration:**

NCT00828997 and EudraCT EU 2007-006539-29.

## Introduction

A population-based surveillance over 4 years after licensure of the 7-valent pneumococcal conjugate vaccine (Prevenar, PCV7) for children in the USA showed a significant decrease of invasive pneumococcal disease (IPD) among adults 50 years and older, but also an increase of IPD caused by serotypes not included in the vaccine [1]. A new pneumococcal conjugate vaccine containing 13 different pneumococcal capsular antigens 1, 3, 4, 5, 6A, 6B, 7F, 9V, 14, 18C, 19A, 19F and 23F has recently been approved by the authorities in USA and Europe for primary and secondary immunization in children. The Centre for Disease Control and Prevention (CDC) Advisory Committee on Immunization Practices recently updated recommendations for pneumococcal vaccination, and these include immunization with a dose of 13-valent pneumococcal conjugate vaccine in adults with diseases requiring immunosuppressive treatments and long-term systemic corticosteroids [2].

Pneumococcal vaccination is strongly encouraged by the European League Against Rheumatism (EULAR) for patients with inflammatory rheumatic diseases [3]. Data on the benefit of pneumococcal conjugate vaccine in immunosuppressed patients with rheumatic disease are scarce. Our group has reported on antibody response following vaccination with PCV7 in patients with rheumatoid arthritis (RA) and spondylarthropathy (SpA) including ankylosing spondylitis and psoriatic arthritis treated with different anti-inflammatory remedies. Methotrexate (MTX), but not anti-TNF drugs, was associated with decreased antibody response [4].

Along with anti-TNF drugs newer treatment modalities have been available for treatment of RA in the last decade. These include a chimeric anti-CD20 monoclonal antibody rituximab, a selective T-cell co-stimulation modulator (abatacept) and a humanized anti-IL-6 receptor monoclonal antibody (tocilizumab). Studies on antibody response following pneumococcal vaccination in patients with established arthritis receiving these treatments are scarce.

The present work is an extension of a report on antibody response following pneumococcal vaccination using 7-valent conjugate vaccine in arthritis patients treated with TNF-inhibitors [4]. The objective of the study was to investigate the immunogenicity and tolerability of the 7-valent pneumococcal conjugate vaccine in patients with established RA treated with biologic remedies other than TNF-inhibitors.

## Methods

RA patients regularly monitored at the Department of Rheumatology, Skåne University Hospital in Lund and Malmö, Sweden, were invited to participate in the study as previously described [4]. The Regional Ethic Review Board at Lund University approved the study (file number 97/2007). The study was conducted as an investigator-driven clinical trial, registered online at EudraCT EU 2007-006539-29 [5] and at NCT00828997, and approved by the Swedish Medical Products Agency (MPA; file number 151: 2007/88047). Informed written consent was obtained from all subjects before study entry. Initially, 505 patients with RA or spondylarthropathy participated in the study [4]. In the extended part of the study, RA patients receiving treatment with biologic remedies other than TNF antagonists were offered vaccination. Only RA patients being on the biologic drug for at least 4 weeks were eligible for the study. The vast majority of these patients had previously been treated with one or more anti-TNF remedies and the number of previously given biologic treatments was calculated. All patients received one dose (0.5 ml) of heptavalent pneumococcal conjugate vaccine (Prevenar) intramuscularly. Blood samples were drawn at vaccination and 4 to 6 weeks thereafter. Immunoglobulin (Ig)G antibodies specific for *Streptococcus pneumoniae* capsular polysaccharides 6B and 23F were measured using ELISA as previously described [6]. Briefly, ELISA plates were coated with the polysaccharides 23F or 6B. Dilutions of human sera absorbed with pneumococcal cell wall polysaccharide were then added to the ELISA plates. A reference serum was included on all plates. The serotype-specific antibodies for 23F and 6B were detected using alkaline phosphatase-conjugated goat anti-human IgG (-chain specific) F(ab’)2 fragments, followed by addition of the substrate *p*-nitrophenyl phosphate. The optical density at 405 nm was measured with an ELISA plate reader. The optical density of the colored end-product is proportional to the amount of specific antibodies present in the serum. The lower limit of detection was 0.02 mg/L for serotype 6B and 0.01 mg/L for 23F.

Geometric mean antibody levels (GML) prior to and after vaccination were calculated from log-transformed values and compared to previously reported GML of MTX-treated patients and controls (SpA patients on non-steroidal anti-inflmmatory drugs (NSAIDs)/analgesics). Antibody response (AR) was defined as the ratio between post- and prevaccination antibody levels and a positive AR (posAR) as at least two-fold increase in AR as described [4].

### Study population

In total, 88 patients (74% women) treated with biologic remedies other than TNF inhibitors participated in the study. Mean (SD; range) age and disease duration in the whole group was 60.2 (2.0; 41.0 to 85.0) and 16.0 (2.0; 2.0 to 46.0) years, respectively. Mean Disease Activity Score (DAS) (SD) and Health Assessment Questionnaire (HAQ) score (SD) at vaccination was 4.5 (0.2) and 1.3 (0.1), respectively. Of these 88 patients, 89% patients were rheumatoid factor (RF)-positive and 82% were anti-cyclic citrullinated peptide (CCP)-positive. All patients fulfilled the American College of Rheumatologists (ACR) classification criteria for RA [7]. Treatment was as follows: 55 patients were treated with rituximab, 17 with abatacept and 16 with tocilizumab. Biological treatment was given as monotherapy to 40 of 88 (44.5%). The remaining 48 patients received concomitant MTX and of these, 3 were also treated with sulphasalazine and 2 with hydroxychloroquine. Mean number (range) of previous biologics was 1.2 (0.0 to 4.0), 2.4 (0.0 to 5.0) and 2.0 (0.0 to 6.0) in patients on rituximab, abatacept and tocilizumab, respectively. Rituximab-treated patients had lower IgG and IgM levels compared to those with the other two treatments.

### Statistical analysis

Non-parametric tests were generally used. Differences in AR between two treatment groups at the time were calculated using the Mann-Whitney *U*-test. The association between age, disease duration, DAS, HAQ score, ongoing MTX dose, ongoing prednisolone dose and IgM/IgG levels at vaccination and positive immune response (posAR; yes/no) was analyzed using the Mann-Whitney *U*-test. The Chi-squared test was applied to calculate the influence of gender, RF/anti-CCP positivity (yes/no), concomitant MTX (yes/no), concomitant prednisolone (yes/no) and for each biologic treatment at vaccination on posAR (yes/no). Possible predictors of posAR were explored in multivariate binary logistic regression model adjusted for significantly different baseline demographic and disease characteristics. Because age and disease duration correlated significantly (Spearman correlation factor >0.3; *P* <0.001), as well as 28-joint DAS (DAS28) and HAQ score (Spearman correlation factor >0.6; *P* <0.001), only disease duration and DAS28 could be included in the same regression analysis. The following variables were included in the final model: disease duration, gender, DAS, concomitant MTX, concomitant prednisolone dose and rituximab treatment at vaccination. Due to the limited number of patients, the impact of abatacept and tocilizumab treatment on posAR could not be analyzed in a multivariate logistic regression model.

## Results

Demographic and disease characteristics at vaccination, and GML of pre- and postvaccination antibody levels for serotype 6B and 23F, for each separate serotype in RA patients treated with rituximab, abatacept and tocilizumab are summarized in Table 1. Previously reported, corresponding data in RA patients on MTX and controls (SpA patients on NSAIDs and/or analgesics) are also shown.

**Table 1:** Demographic and disease characteristics at vaccination, and prevaccination- and postvaccination GML in RA patients on different treatments and controls

**Treatment groups**	**Rituximab monotherapy**	**Rituximab + MTX**	**Abatacept (n = 17)**	**Tocilizumab (n = 16)**	**MTX**	**Controls**
					**(n = 85)**	**(n = 86)**
	**(n = 29)**	**(n = 26)**				
**Female gender, %**	72%	62%	88%	81%	79%	45%
**Age, years, mean (SD) (range)**	68.9 (9) (50 to 87)	59.9 (12) (39 to 85)	56.6 (13) (29 to 75)	55.6 (12) (27 to 72)	61.5 (14) (24 to 85)	51.6 (12) (22 to 75)
**Disease duration, years mean (SD) (range)**	21.3 (14) (0 to 57)	14.5 (12) (2 to 50)	14.9 (2.9) (2 to 46)	13.8 (12) (0 to 43)	11.4 (10) (0 to 40)	12.7 (12) (0 to 45)
**HAQ, 0 to 3**	1.2 (0.8)	1.1 (0.6)	1.4 (0.6)	1.5 (0.7)	0.7 (0.6)	0.5 (0.5)
**DAS, 0 to 7**	3.9 (1.2)	3.9 (1.1)	4.9 (1.2)	3.6 (1.7)	3.7 (1.2)	3.0 (1.1)
**Ongoing MTX, yes, %**	----	100%	76%	56%	100%	----
**MTX mg/week, mean**	----	17.2	15.6	14.7	16.4	----
**Ongoing prednisolone,** **yes, %**	55.2%	65.4%	70.0%	53.3%	31.3%	6.2%
**Prednisolone, mg/week, mean**	30.9	35.4	32.7	27.5	14.0%	2.2
**CRP mg/L, mean**	15.8	6.4	9.7	3.9	8.9	7.7
**TJC28, mean (range)**	3.5 (0 to 23)	4.7 (0 to 19)	8.2 (0 to 20)	7.6 (0 to 28)	2.8 (0 to 12)	1.9 (0 to 18)
**SJC, mean (range)**	3.4 (0 to 15)	3.9 (0 to 13)	4.8 (0 to 12)	5.1 (0 to 25)	2.0 (0 to 11)	0.4 (0 to 5)
**RF-positive, %**	93%	89%	88%	81%	78%	NA
**Anti-CCP-positive, %**	86%	92%	82%	63%	75%	NA
**Smoker, %**	14%	31%	19%	14%	19%	12%
**IgG at vaccination, g/L, mean (SD)**	8.2 (2.9)	8.0 (2)	9.1 (1.9)	8.7 (2.6)	9.7 (2.5)	9.9 (2.1)
**IgM at vaccination, g/L, mean (SD)**	0.8 (0.7)	0.9 (0.5)	1.3 (0.7)	1.1 (0.5)	1.1 (0.6)	1.0 (0.6)
**IgA at vaccination, g/L, mean (SD)**	2.3 (1.3)	2.4 (0.9)	2.9 (1.3)	2.5 (0.9)	2.5 (1.1)	2.5 (1.1)
**Pneumococcal vaccine previously, %**	24.1%	23.1%	11.8%	6.3%	10.6%	1.2%
**Last biologic treatment before vaccination, days, mean (range)**	71 (0 to 501)	102 (0 to 894)	8 (0 to 26)	7 (0 to 34)	NA	NA
**Prevaccination antibody levels for 6B, mg/L, GML (95% CI)**	0.3 (0.2-0.6)	0.4 (0.2, 0.8)	0.6 (0.3, 1.4)	0.4 (1.0, 1)	2.0 (1.4, 2.8)	2.9 (2.1, 4.0)
**Postvaccination antibody levels for 6B, mg/L, GML (95% CI)**	0.4 (0.2-0.8)	0.4 (0.2, 0.8)	1.1 (0.4, 2.7)	1.7 (0.5, 5.8)	3.5 (2.5, 4.9)	9.5 (6.7, 13.6)
**Prevaccination antibody levels for 23F, mg/L, GML (95% CI)**	0.2 (0.1, 0.4)	0.3 (0.1, 0.5)	0.4 (0.2, 0.9)	0.2 (0.1, 0.5)	0.7 (0.5, 1.1)	0.97 (0.7, 1.4)
**Postvaccination antibody levels for 23F, mg/L, GML (95% CI)**	0.3 (0.2, 0.6)	0.4 (0.2, 0.8)	1.1 (0.6, 2.3)	2.2 (0.9-5.3)	1.9 (1.4, 2.6)	6.4 (4.5, 9.1)

Controls were patients with spondyloarthropathy on non-steroidal anti-inflammatory drugs/analgesics. GML, geometric mean antibody levels; RA, rheumatoid arthritis; MXT**,** methotrexate; HAQ, health assessment questionnaire; DAS, disease activity score; TJC, tender joint count; SJC, swollen joint count; RF, rheumatoid factor; anti-CCP, anti-cyclic citrullinated peptide antibody; NA, not applicable.

**Figure 1:**
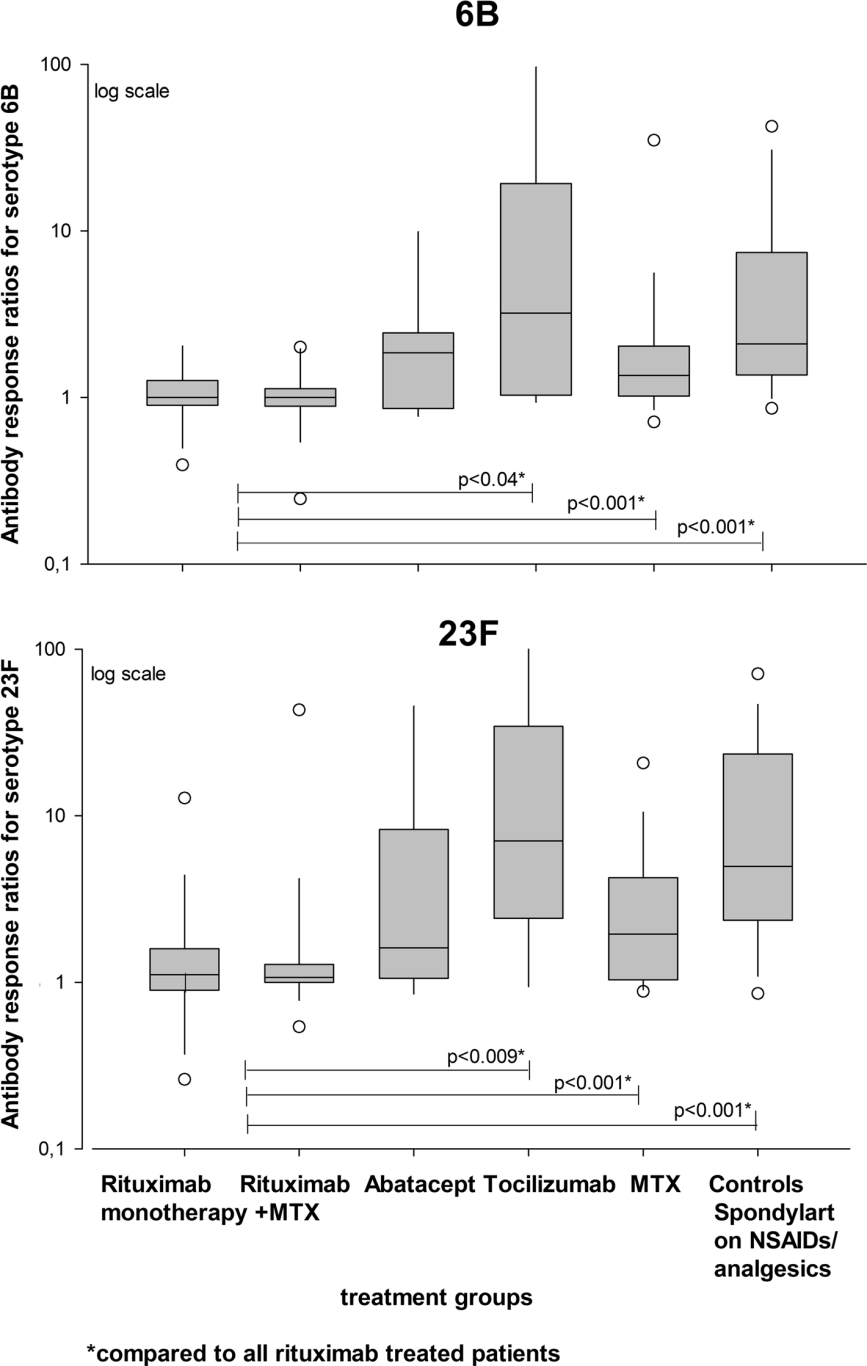
**Box plots show 5th/95th percentiles of antibody response, that is, ratio of postvaccination/prevaccination antibody levels for 6B and 23F in different treatment groups.** Statistically significant differences in antibody response for each serotype when two different treatment groups were compared (Mann-Whitney *U*-test) are indicated. MTX, methotrexate; Spondylart, spondyloarthritis; NSAID, non-steroidal anti-inflammatory drug.

Figure 1 shows box plots with AR for serotype 6B and 23F in the different treatment groups.

### Rituximab

At vaccination mean rituximab treatment time was 1.3 years. Of 55 patients treated with rituximab, 26 (46%) were on concomitant MTX. The differences in AR, that is, post-/prevaccination antibody levels between patients on rituximab as monotherapy or rituximab + MTX were not significant for any of the serotypes. Rituximab-treated patients as a group had significantly lower AR for each serotype compared to RA patients on MTX, RA on tocilizumab, and controls (Figure 1). Compared to abatacept, rituximab-treated patients had lower AR for each serotype but differences were not significant.

**Figure 2:**
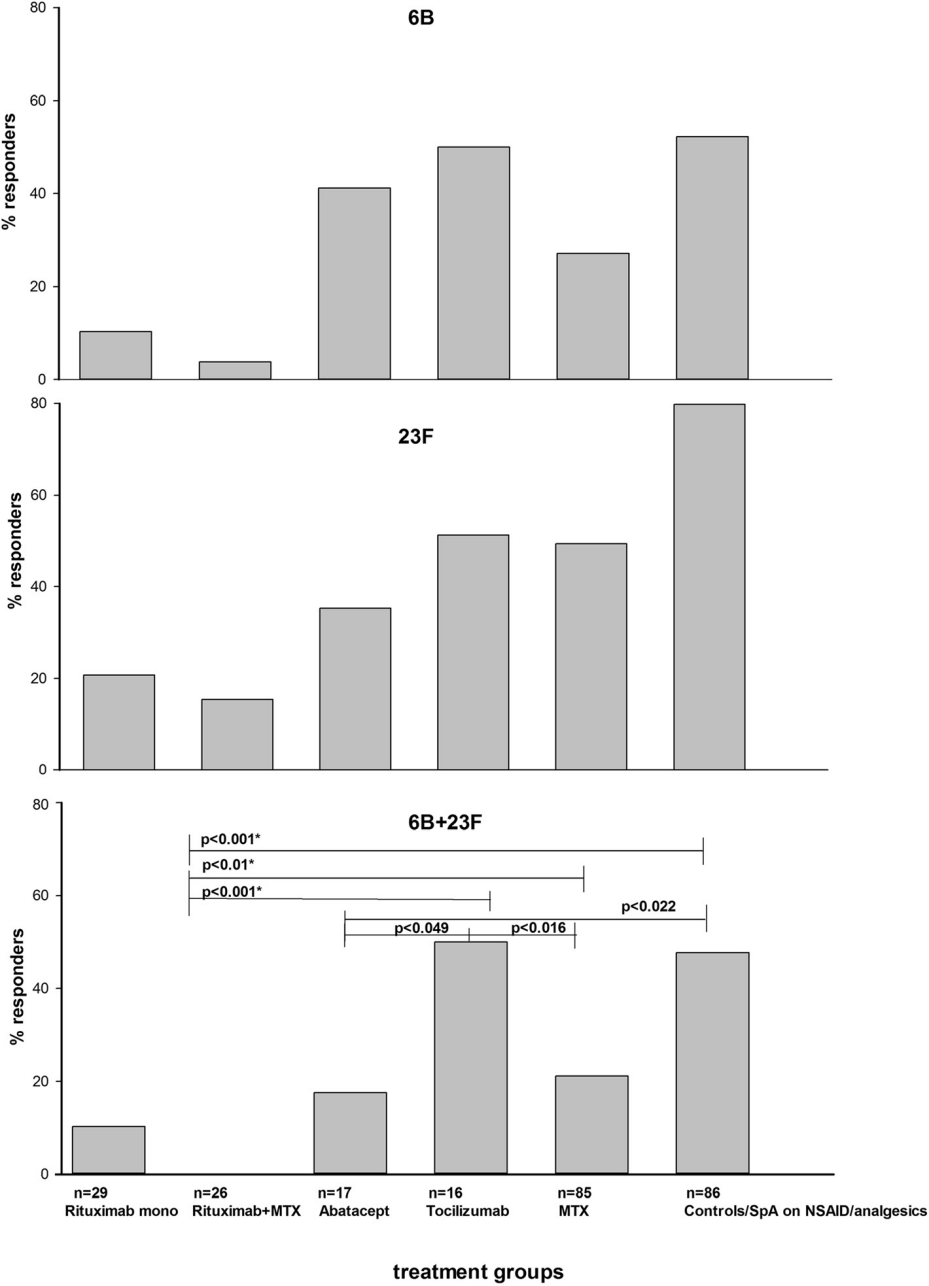
**Percentage of patients with positive immune response defined as at least two-fold increase in prevaccination antibody levels for serotype 6B, 23F and both 6B and 23F in different treatment groups.** Statistically significant differences in positive immune response for both 23F and 6B serotypes when two different treatment groups were compared to each other (Chi-squared test) are indicated. MTX, methotrexate; SpA, spondyloarthritis; NSAID, non-steroidal anti-inflammatory drug.

Rituximab-treated patients as a group had impaired posAR for both serotypes tested compared to MTX, tocilizumab and controls (Figure 2) but not abatacept-treated patients with RA (*P* = 0.112). More patients treated with rituximab as monotherapy were responders for each separate serotype but the differences were not significant. However, no patients on rituximab + MTX were responders for both serotypes together (Figure 2).

All patients treated with rituximab (n = 55) had received a minimum of two treatments and vaccination was administered at least 4 weeks after the first treatment. Mean (range) time between last treatment and vaccination was 86 (0 to 894) days. Of 55 patients 37 (67.3%) were vaccinated within 3 months and the majority of patients (n = 49; 89.1%) within 6 months following the last rituximab infusion. There were no statistically significant differences in antibody responses between patients vaccinated within ≤180 days compared to those receiving vaccination >180 days after the last rituximab treatment.

### Abatacept

Of 17 patients treated with abatacept, 13 patients (76%) received concomitant MTX. Vaccination was administrated on average 8 (0 to 26) days after infusion. Mean abatacept treatment time at vaccination was 0.8 years. Demographic and disease characteristics did not differ significantly between patients receiving abatacept as monotherapy or combined with MTX. All patients treated with abatacept were analyzed as a group. Compared to MTX-treated patients with RA, abatacept-treated patients had better posAR for serotype 6B but worse for serotype 23F. The proportion of patients on abatacept with posAR for both serotypes was lower compared to RA patients on MTX but the differences were not significant. A significantly smaller proportion of patients on abatacept had posAR for both serotypes compared to those on tocilizumab and controls (Figure 2).

### Tocilizumab

Of 16 patients on tocilizumab, 9 (54%) had concomitant MTX. Vaccination was performed on average (range) 7 (0 to 34) days after infusion. Mean tocilizumab treatment time at vaccination was 0.3 years. Regardless of MTX treatment, tocilizumab-treated patients as a group had lower pre- and postvaccination GML levels for both serotypes compared to controls. However, the immune response for each serotype, and posAR for 6B and 23F both separately and together were as good as those of controls. Tocilizumab-treated patients had significantly better posAR compared to rituximab-, abatacept- or MTX-treated patients with RA (Figure 2).

### Predictor analysis

Possible predictors of posAR for both serotypes were studied using a logistic regression model with adjustment for differences in disease duration, gender, DAS, concomitant MTX and prednisolone at vaccination (Table 2). Both rituximab and MTX predicted diminished posAR for both serotypes (*P* = 0.033 and 0.044, respectively). Patients with longer disease duration (*P* = 0.016) and higher disease activity (*P* = 0.028) showed decreased response (*P* = 0.07), but no significant effect of sex or concomitant prednisolone dose was observed.

**Table 2:** Predictors of positive immune response for both serotypes (23F + 6B) using multivariate logistic regression analysis

	***P*-value**	**Odds ratio**	**95% CI**
Sex, male/female	0.269	0.34	0.05, 0.31
Disease duration, years	0.016	0.90	0.82, 0.98
DAS28	0.028	0.53	0.30, 0.93
MTX at vaccination, yes/no	0.044	0.13	0.02, 0.94
Prednisolone at vaccination, mg/week	0.118	0.02	1.00, 1.04
Rituximab at vaccination, yes/no	0.033	0.06	0.01, 0.80

DAS28, 28-joint Disease Activity Score; MTX, methotrexate.

As previously reported, pneumococcal conjugate vaccine was generally well-tolerated and no severe adverse event was reported within 4 to 6 weeks after vaccination among patients receiving biologic treatment other than anti-TNF. Pain at the injection site was the most commonly reported side effect, followed by headache, fever and fatigue for a few days. One case of uncomplicated lower urinary tract infection requiring treatment with antibiotics was reported in a patient on rituximab. One patient suffered from significantly more joint pain but the number of swollen joins was unchanged compared to that before vaccination. No increased activity in RA disease or onset of other immunological disease was observed.

## Discussion

Here we report a study on AR following pneumococcal vaccination using pneumococcal conjugate vaccine in RA patients treated with biologic remedies other than TNF inhibitors.

In this cohort of patients with established RA, who were previously exposed to up to six other biologic drugs, ongoing treatment with abatacept and rituximab impaired the antibody response. The negative impact was most pronounced in rituximab-treated patients and additionally diminished when concomitant MTX was used. AR in patients on tocilizumab was as good as that of arthritis patients not receiving immunosuppressive drugs.

T-cell-dependent mechanisms are important for AR following immunization with protein antigen [8]. Antigen-presenting cells, including B-cells, are required for presentation of protein antigens to naive T-cells which thereby stimulate secretion of different cytokines and activate B-cells to differentiate into antigen-specific Ig-producing plasma cells. A lack of mature B-cells results in a lower amount of plasma cells and as a consequence lower production of serotype-specific antibodies [8]. As pneumococcal conjugate vaccine consists of capsular polysaccharides conjugated to a protein carrier, this could be one possible mechanism for reduced AR following B-cell depletion therapy using rituximab. Our results are in line with previously reported diminished IgG response following immunization with protein antigen (influenza vaccination) in RA patients on rituximab [9]. A randomized controlled study investigating AR following pneumococcal vaccination using 23-valent polysaccharide vaccine also showed impaired response in RA patients on rituximab [10].

Our findings of modestly decreased levels of circulating IgG, IgM and IgA among rituximab treated patients are in line with those previously reported in RA patients after subsequent rituximab courses [11]. Indeed, patients participating in this study had received at least two rituximab treatments at the time of vaccination and a substantial number was treated with subsequent courses every 6 months. Thus, low levels of serotype-specific antibodies already present before vaccination might be explained by universal depletion of B-memory cells. In contrast to results from van Assen *et al*. no significant correlation between timing of vaccination in relation to administration of rituximab was found in our study [9]. Long-lasting depletion of the whole pool of B-memory cells and not only circulating B-cells caused by repeated rituximab treatments might explain the diverging results.

The abatacept-treated RA patients had decreased antibody response compared to controls and tocilizumab-treated patients with RA. Abatacept attenuates activation of naive T-cells by blocking the interaction between CD80/86 and CD28, a co-stimulation signal required for full T-cell activation. RA patients have many CD28-null T-cells. In addition, abatacept (CTLA-4Ig) also prevents CTLA-4 binding to its ligand [12]. This may result in inhibition of T-cell proliferation and subsequently inadequate stimulation of B-cells. Diminished B-cell immunological response might be a consequence of inadequate stimulation needed for their differentiation into plasma cells. Results from our study are in accordance with another study investigating AR following pneumococcal vaccination after administration of abatacept to healthy adults, where a diminished AR in subjects immunized after administration of one dose of abatacept was reported [12]. The negative impact of abatacept on AR was significantly less prominent compared to that of rituximab. Despite the limited number of abatacept-treated patients participating in the study our results suggest that the inhibition of the secondary co-stimulation signal does not appear to have a critical role in antibody production following conjugated polysaccharide-protein antigen challenge. On the other hand, AR after influenza vaccination in RA patients on abatacept was found to be severely impaired [13].

In contrast to the effects of rituximab and abatacept on AR, we previously reported that the response in arthritis patients on anti-TNF treatments was not significantly different from that in arthritis patients not receiving immunosuppressive drugs immunized with pneumococcal conjugate vaccine, or healthy individuals immunized with polysaccharide vaccine [4,14]. These results are in line with earlier experimental data published by *Cope et al.* showing that anti-TNF treatment in RA did not impair, but enhanced T-cell responses to polypeptide antigen towards normal response [15].

IL-6 plays an important role in differentiation of B-cells into antibody producing plasma cells [16]. Thus blockade of IL-6 would be expected to diminish antibody production. However, antibody response after influenza vaccination was not hampered in patients with RA treated with tocilizumab [17]. Antibody response among patients treated with tocilizumab in our study, immunized after an average of 7 days following the last treatment course, was similar to controls (SpA patients not treated with immunosuppressive drugs). Along with results from Mori *et al*. our results suggest that IL-6 is not essential for antibody production after conjugated polysaccharide-protein challenge, which is consistent with previously reported experimental data [17,18]. IL-6 may still have an impact on IgG production by influencing IgG subclasses differently, but this is not possible to assess from our results.

Ongoing rituximab treatment was identified as a predictor of insufficient AR. Patients with higher disease activity and longer disease duration had decreased response but no effect of age and sex was observed. The majority of patients enrolled in the present study were women and differences in age between treatment groups were rather small. Thus the impact of age and sex might be difficult to discern.

MTX was identified as a predictor of impaired positive AR in a multivariate logistic regression model, which is in accordance with our previous reports including arthritis patients treated by anti-TNF remedies [4,14]. The number of patients treated with abatacept and tocilizumab was limited in the present study, precluding the separate analysis of effect of MTX on AR in these groups. Among rituximab-treated patients those receiving concomitant MTX had a lower antibody response, which also confirms our previous results [4,14].

The AR is a surrogate marker of protection against infection. The correlation between certain antibody levels and protection has been reported for several vaccines, including pneumococcal vaccine [19]. However, results from the present study do not exclude the possibility that rituximab- and abatacept-treated patients would mount the appropriate immune response when exposed to *Streptococcus pneumoniae.*

In addition, antibody response to two of seven serotypes included in the vaccine was studied. Serotypes 6B and 23F are shown to be associated with invasive pneumococcal disease in Sweden, which was the reason for choosing these serotypes [20]. Although response to other serotypes may differ, we hypothesize that the effects of different biologic treatments would influence AR to other serotypes in a similar fashion.

The disadvantage of the present study is the small sample size in groups treated with abatacept and tocilizumab compared to RA patients on MTX, and controls. Further research including larger groups of patients on newer biologics given as monotherapy and in combination with conventional disease-modifying anti-rheumatic drugs is needed.

## Conclusions

In this cohort of patients with established RA, treatment with rituximab and abatacept was associated with impaired antibody response following protein-polysaccharide antigen challenge, but the impact of rituximab was more substantial. The IL-6 receptor blockade did not hamper antibody response in these patients. Pneumococcal vaccination should preferably be encouraged before initiation of rituximab or abatacept treatment. Pneumococcal vaccine administered during ongoing anti-IL-6 receptor treatment seems to be associated with sufficient immune response in patients with RA.

## Abbreviations

Anti-CCP: Anti-cyclic citrullinated peptide; AR: Antibody response; DAS: Disease activity score; GML: Geometric mean antibody levels; HAQ: Health assessment questionnaire; Ig: Immunoglobulin; IL: Interleukin; IPD: Invasive pneumococcal disease; MTX: Methotrexate; NSAID: Non-steroidal anti-inflammatory drug; posAR: Positive antibody response; RA: Rheumatoid arthritis; SpA: Spondylarthropathy; TNF: Tumor necrosis factor.

## Competing interests

Prevenar® vaccine for this study was provided by Wyeth Pharmaceuticals Philadelphia, Pennsylvania, U.S. All authors declare no competing interest.

## Authors’ contributions

MCK participated in the design of the study, performed the statistical analysis and wrote the manuscript. TS, LT, GJ and PG conceived of the study, participated in its design and coordination and helped to draft the manuscript. All authors read and approved the final manuscript.
